# The Involvement of the European Master in Disaster Medicine (EMDM) Alumni in the COVID-19 Pandemic Response: An Example of the Perceived Relevance of Disaster Medicine Education during Disasters

**DOI:** 10.1017/S1049023X22001340

**Published:** 2022-09-15

**Authors:** Awsan A.S. Bahattab, Monica Linty, Ives Hubloue, Michel Debacker, Francesco Della Corte, Luca Ragazzoni

**Affiliations:** 1. CRIMEDIM –Center for Research and Training in Disaster Medicine, Università del Piemonte Orientale (UPO), Humanitarian Aid and Global Health, Via Lanino, Novara, Italy; 2. ReGEDiM –Research Group on Emergency and Disaster Medicine, Vrije Universiteit Brussel (VUB), Brussels, Belgium

**Keywords:** COVID-19, curriculum, disaster medicine, medical education, pandemics

## Abstract

**Introduction:**

The coronavirus disease 2019 (COVID-19) pandemic has revealed a gap in disaster preparedness of health workers globally. Disaster medicine education is a key element to fill this gap.

**Objectives:**

This study evaluated the involvement of the European Master in Disaster Medicine (EMDM) Alumni in the current COVID-19 pandemic response and their self-perceived value of the EMDM educational program in accomplishing their tasks during the disaster.

**Methods:**

An online survey targeting the EMDM Alumni was conducted from January through March 2021. Quantitative data were described using percentages or means, as appropriate, while qualitative data were categorized using deductive thematic analysis.

**Results:**

In total, 259 Alumni completed the survey. Most of the Alumni (88.03%; standard error of the proportion [SEp] = 0.02) participated directly in the COVID-19 pandemic response – nationally or internationally – with different roles and responsibilities at different levels and sectors. Around 25% of the Alumni reported an increase in their tasks and responsibilities due to COVID-19 response, but few worked beyond their main specialization (5.26%) or expertise (2.19%). Moreover, Alumni shifted their role from clinical practice to managerial, public health, education and training, and policymaking roles during COVID-19 (*P* <.001). Participants believed that the EMDM study program and the competencies acquired during the course were relevant and useful to perform their tasks during the COVID-19 pandemic response (mean = 5.26; 5.17 standard error of the mean [SEM] = 0.108, 0.107), respectively. Around 36% (SEp = 0.03) of the participants deemed that some contents were not sufficient for COVID-19 response.

**Conclusion:**

Most of the EMDM Alumni were involved in the COVID-19 pandemic response, playing diverse roles with an increased level of responsibility compared to those played before the pandemic. Moreover, the Alumni perceived the EMDM curriculum as relevant for accomplishing their tasks. However, they also reported gaps within the curriculum, especially topics related to outbreak and pandemic response. The findings of the study stress the value of investing in disaster medicine education world-wide and of pushing to update and standardize post-graduate disaster medicine curricula.

## Introduction

The frequency, scale, and impact of disasters have increased significantly over the last decades.^1^ In the last two years, the coronavirus disease 2019 (COVID-19) pandemic alone claimed more than six million lives, resulted in more than 525 million confirmed cases, and stressed the health systems world-wide.^2^

A quality and timely disaster response requires a competent health workforce at all levels. The Sendai Framework for Disaster Risk Reduction 2015-2030 sets the training of workforce in disaster medicine among the priorities of action for effective disaster preparedness.^3^ Likewise, the Health Emergency and Disaster Risk Management (H-EDRM) framework of the World Health Organization (WHO; Geneva, Switzerland) underscores the role of the H-EDRM workforce in building resilient health systems.^4^ Yet, the current COVID-19 pandemic reveals that many health workers were poorly trained in emergency preparedness and disaster management at different levels.^5,6^ Hence, the COVID-19 pandemic has not only reaffirmed the necessity of basic training in disaster medicine,^7–10^ but has also stressed the need for advanced training in the form of post-graduate higher education in disaster medicine.^11^

Insufficiencies were evident in the existing training and educational programs of disaster medicine in terms of availability, geographical distribution, and topics covered.^12^ Investment in building the capacity by developing blended programs that use innovative teaching methods was recommended to fill the current gaps in disaster medicine education.^13^

The European Master of Science in Disaster Medicine (EMDM), a multidisciplinary advanced master program, aims to address the gap in the education and training of the health workforce through the development of a disaster medicine study program at the academic level.^14^ The program is jointly organized by the Center for Research and Training in Disaster Medicine, Humanitarian Aid, and Global Health (CRIMEDIM) of the Università del Piemonte Orientale (Novara, Italy) and the Research Group on Emergency and Disaster Medicine (ReGEDiM) of the Vrije Universiteit Brussel (Brussels, Belgium). The EMDM is a one-year program of nine faculty-guided e-learning units, two-weeks face-to-face residential training, and scientific thesis writing and defense.^14^ The competency-based curriculum of the EMDM is in line with the recommendations of the World Association for Disaster and Emergency Medicine (WADEM; Madison, Wisconsin USA) and the International Society of Disaster Medicine (Geneva, Switzerland), which defined the four main topics of disaster medicine education as medical care, public health, disaster management, and education and training.^15,16^

In the period from 2000 through 2021, 621 students from 89 countries around the world were enrolled with a maximum of 35 students per academic year. The didactic concept is based on the application of a competency-based, multidisciplinary, and collaborative approach, evidence-based practices, and a number of innovative educational methods such as experiential learning through table-top and virtual reality simulations, drills, and full-scale exercises.^17,18^

Currently, the EMDM Alumni represent an example of a trained health workforce spread world-wide. The impact of the EMDM in the Alumni’ professional career in the field of disaster medicine and their participation in disaster preparedness and response programs at national and international levels have already been explored.^19^ However, the involvement of the EMDM Alumni during outbreak and pandemic responses was not reported previously. Most importantly, the unprecedented risk, scale, and impact of the COVID-19 pandemic pose a new perspective for disaster management.^20^ This should be accompanied by updating the disaster medicine curriculum to allow trainees to manage future disasters.^8^

This study, therefore, explored the involvement of the EMDM Alumni in the COVID-19 pandemic response and their self-perceived relevance of the EMDM educational program to perform their tasks during the current global disaster. The findings might help policy makers, disaster medicine professionals, and scholars to recognize the importance of investing in disaster medicine education and evaluating possible gaps in current disaster medicine curriculum frameworks world-wide.

## Methods

### Study Design and Study Participants

A cross-sectional online survey targeted all the 576 students who had been enrolled in the EMDM program in the years 2000/2001–2019/2020. The study participants were recruited through the list of all Alumni and contacted with their contact e-mail available with the faculty. Thus, all Alumni have the same probability to participate in the study, then each Alumni can either respond or not.^21^ At the time of the data collection, the students enrolled in 2020/2021 were only in the second month of a one-year program, hence excluded.

### Study Tool

The data were collected using an online questionnaire with closed- and open-ended questions (Supplementary Material I; available online only). The study objectives guided the questionnaire development. The questionnaire first was reviewed for content validity by experts in disaster medicine training and was tested with small number of the study population (n = 15). The questionnaire had 27 questions and was divided into three main sections to collect the following information:

1. Alumni Background: country of residence, age, sex, graduation, and employment before COVID-19 response (sector, provincial, national, or international level, and role).
2. Alumni Role during COVID-19 Pandemic: country of response, change in employment during the response (sector, level, and role), work setting, degree of decision making, and the barriers if not participated in the COVID-19 pandemic response.
3. Alumni Self-Perceived Relevance and Gaps of the EMDM Educational Program to Perform their Tasks during the COVID-19 Pandemic Response: the scale of the Alumni-perceived relevance of EMDM educational program for the COVID-19 response reported on one-to-seven numerical rating (interval) scale, comprising of nine items. A reliability analysis check was carried out on the scale and showed excellent internal consistency (Cronbach’s α = 0.95).^22^

### Data Collection

The anonymous survey was set up in the SurveyMonkey (San Mateo, California USA) Software and an invitation to participate in the study was sent via e-mail to 516 Alumni as no up-to-date e-mail addresses were available for 60 Alumni. The survey was opened on January 25, 2021 and was made available for six weeks (until March 6, 2021). Reminder emails were sent at the third and the sixth weeks of the survey.

### Analysis

After the survey closed, all data were downloaded into a Microsoft Excel (Microsoft Corp.; Redmond, Washington USA) spreadsheet and cleaned. The quantitative data were described using percentage for categorical data or mean for continuous data and intervals, including numerical rating scale.^23^ The 95% confidence interval (95%CI) were reported for continuous data and proportions with binomial distribution. The analysis of each question was based on the total number of respondents per question. The marginal homogeneity test was used to assess if a statistically significant change has occurred in the Alumni employment sector, employment level, or their professional role during the COVID-19 response. To examine the relevance of the overall EMDM program, one sample t-test was used to compare the mean score of the relevance of EMDM educational program with the arithmetic mean of the scale. The tests were performed with IBM SPSS version 28 (IBM Corp.; Armonk, New York USA). Significant results were defined as P <.05. Open questions’ responses were analyzed using deductive thematic analysis and categorized under the EMDM course units and specific topics, as appropriate.

### Ethical Consideration

As all data were identified and reported in aggregate, the local Ethics Committee at the Università del Piemonte Orientale, Novara, Italy deemed the study (protocol number: RQ29820) exempt from institutional review approval. The participants were requested to consent to participate before proceeding with the questionnaire.

## Results

Among the 516 sent e-mails, 49 e-mails were bounced back. The response rate was 55.46% (95% CI = 50.9-59.9%; n = 259/467) and the survey completion rate was 85.76% (95% CI = 81.5-89.4%; n = 259/302).

### Alumni Background

Table 1 reports the data regarding EMDM Alumni’ background before the outbreak of COVID-19. At the time of the survey, 255 respondents were living in 65 different countries on six continents.

**Table 1.** EMDM Alumni’ Background (n = 259)

Variable	Mean (95% CI)	
***Age (n = 251)***	46.94 (45.77-48.11)	
***Sex (n = 256)***	**No.**	**% (95% CI)**
Male	185	72.27% (66.6-76.5%)
Female	71	27.73% (22.5-33.4%)
***Graduates (n = 259)***		
Yes	226	87.26% (82.8-90.9%)
**Employment and Professional Background Prior to COVID-19 Pandemic (n = 259)**		
***Sector of Main Employment***	**No.**	**%**
Health Services Delivery (primary, secondary, tertiary, specialized)	159	61.39%
Governmental Health Agency (ministry of health, regional/district health office)	45	17.37%
Research/Higher Education Institution	21	8.11%
United Nations/Nongovernmental Organization (national/international)	18	6.95%
Other	16	6.18%
***Level of Main Employment***		
International Level	28	10.81%
National Level	73	28.19%
Regional/Provincial Level	93	35.91%
District Level	49	18.92%
Other	16	6.18%
***Main Professional Role***		
Physician	162	62.55%
Managerial Role	35	13.51%
Medical Education/Training	17	6.56%
Public Health Professional	14	5.41%
Nurse/Allied Health Professional	9	3.47%
Research	6	2.32%
Policymaking Role	3	1.16%
Other	13	5.02%

Abbreviations: COVID-19, coronavirus disease 2019; EMDM, European Master in Disaster Medicine.

Referring to the main role of the participants, most of the respondents were physicians (62.55%; n = 162/259) while 13.51% held a managerial role (n = 35/259) and 8.88% worked in an academic environment (n = 22/259). The respondents were working at the national (28.19%; n = 73/259) or provincial (35.91%; n = 93/259) levels, and mainly for health services delivery (61.39%; n = 159/259) and governmental health agencies (17.37%; n = 45/259).

### Alumni Response to COVID-19 Pandemic

Most of the Alumni participated in the COVID-19 response (88.03%; 95% CI = 83.7-91.6%); n = 228/259). Among those who were not involved, organizational and/or administrative decisions were the most common reasons reported by the respondents for not being involved (45.61%; n = 14/31), while three participants did not participate due to a self-perceived lack of competencies.

**Figure 1.**
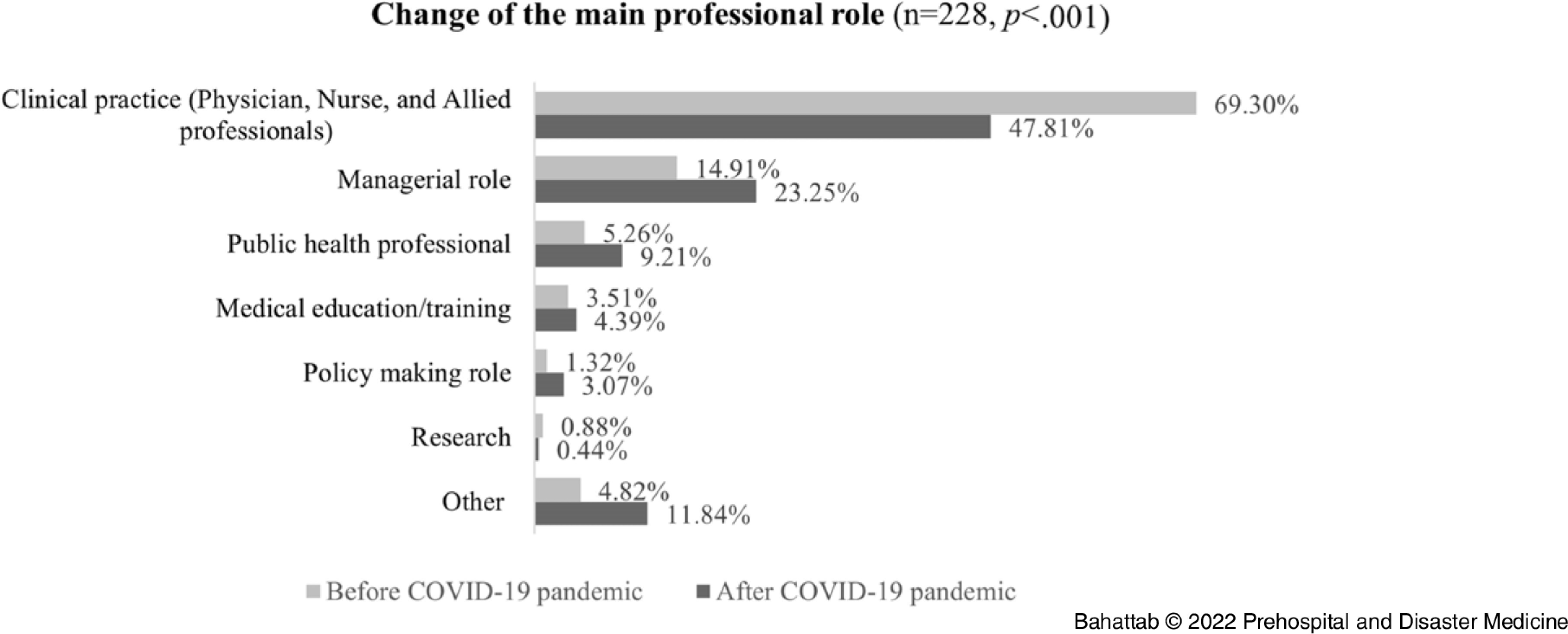
Change of the Main Professional Role. Abbreviation: COVID-19, coronavirus disease 2019.

The Alumni responded to the COVID-19 pandemic in at least 69 countries around the globe, with the 5.75% of them in more than one country (n = 13/226), including a regional (n = 2/226) or a global response (n = 3/226). Most respondents worked in hospital emergency departments (42.98%; n = 98/228) or prehospital settings (12.28%; n = 28/228). While many of the respondents provided direct clinical care to COVID-19 patients (47.81%; n = 109/228), others participated in management (23.25%; n = 53/228), public health activities (9.21%; n = 21/228), policymaking (7.07%; n = 7/228), or education and training (4.39%; n = 10/228); Figure 1.

Approximately 32% (95% CI = 26.2-38.3%; n = 73/228) of the respondents changed their role (Figure 1) during the pandemic through increasing the level of tasks and responsibilities (24.56%; n = 56/228), and, to a lesser extent, by working beyond their main specialization (5.26%; n = 12/228) or expertise (2.19%; n = 5/228). Increasing levels of tasks and responsibilities were reported by all professional categories. Moreover, the change of the Alumni’ roles significantly shifted from clinical practice to managerial, public health, and policymaking roles (P <.001). The change of the Alumni’ roles significantly shifted from clinical practice to managerial, public health, training and education, and policymaking roles. This change, that comprises the sum of all the categories, was statistically significant (P <.001; Figure 1).

**Figure 2.**
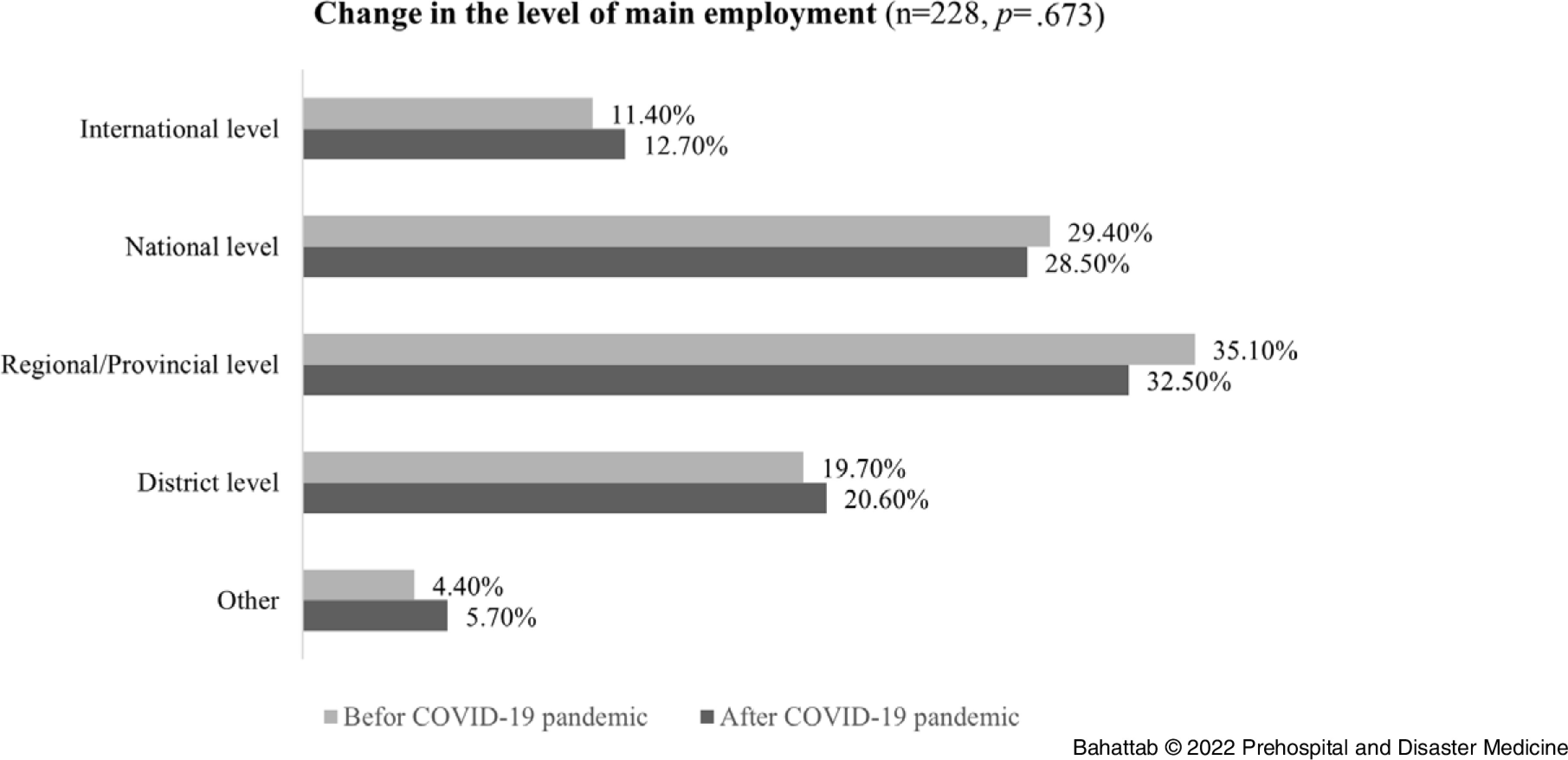
Change in the Level of Main Employment. Abbreviation: COVID-19, coronavirus disease 2019.

**Figure 3.**
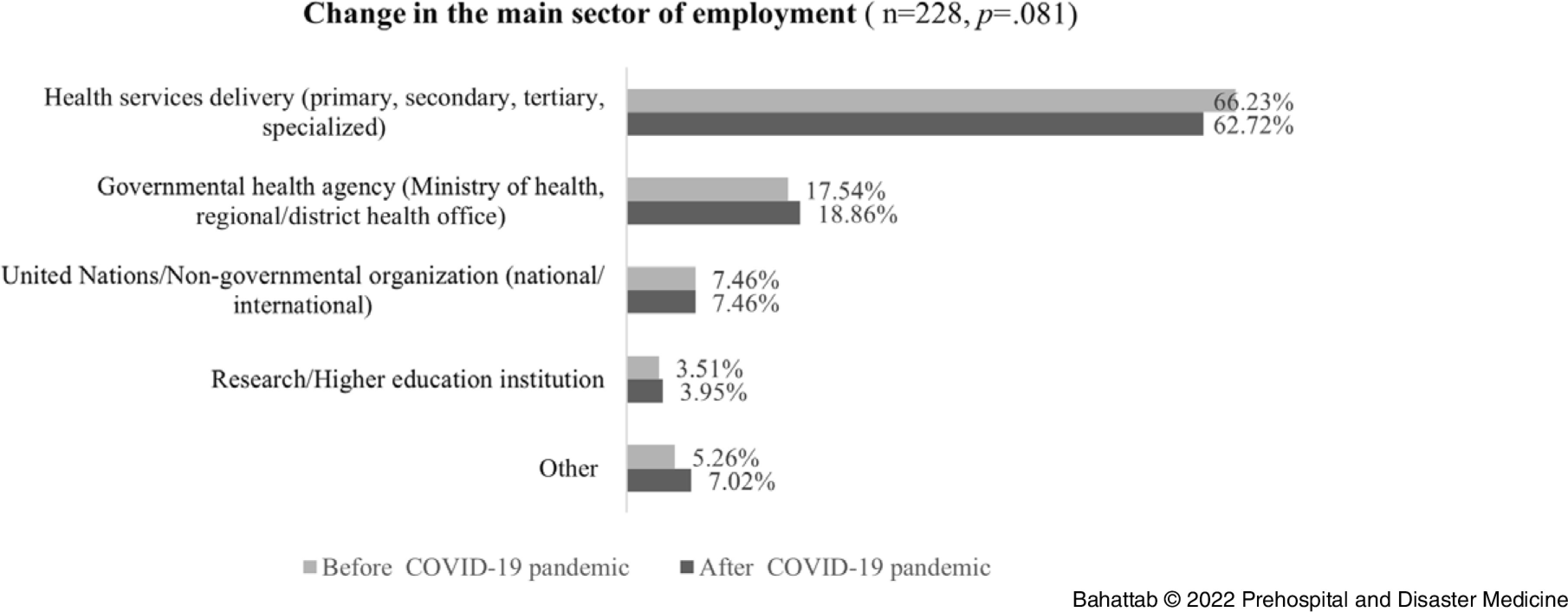
Change in the Main Sector of Employment. Abbreviation: COVID-19, coronavirus disease 2019.

In addition, 13.16% (95% CI = 9.2-18%; n = 30/228) and 10.09% (95% CI = 6.7-14.5%; n = 23/228) of the Alumni changed their employment level and sector, respectively. The Alumni also moved to district and international levels and from health service delivery to governmental agencies and research institutes. However, none of these changes in the employment level and sector were statistically significant (*P* = .673 and .081, respectively; Figure 2 and Figure 3).

The level of decision making also varied among the respondents, but most of them retained a high level of decision making by being able to take decisions at the organizational level (32.89%; n = 75/288), unit (33.77%; n = 77/288), or within small teams (19.30%; n = 44/288), while fewer participants reported that they were making decisions on their personal activities/operations (9.21%; n = 21/288) or taking instruction from others (4.82%; n = 11/288).

### Relevance of EMDM Educational Program for the COVID-19 Response

**Figure 4.**
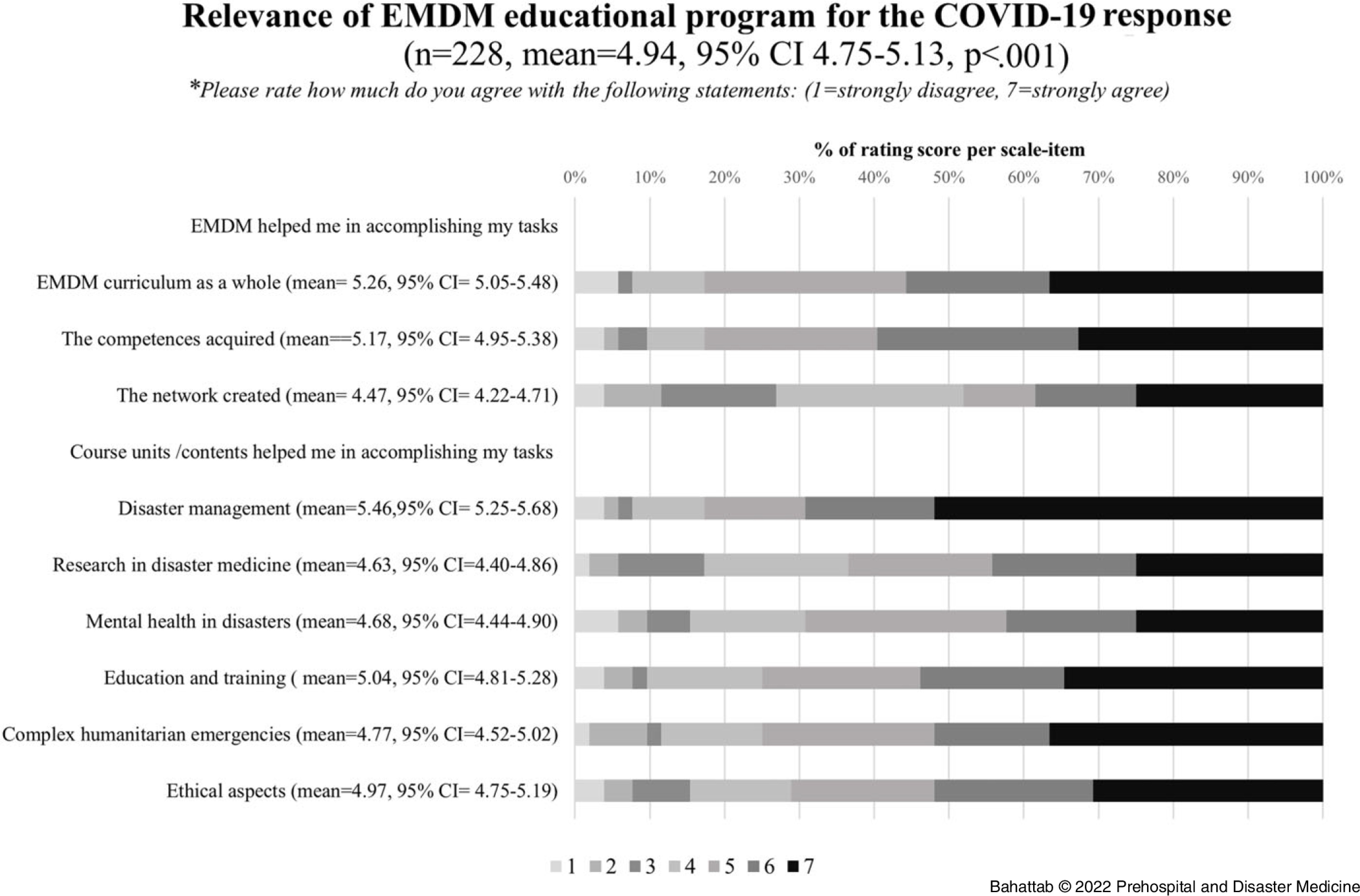
Relevance of EMDM Educational Program for the COVID-19 Response. Abbreviations: COVID-19, coronavirus disease 2019; EMDM, European Master in Disaster Medicine.

On a one-to-seven numerical rating scale, the mean rating of respondents for the overall EMDM program relevance was 4.94 (95% CI 4.75-5.13; P <.001); Figure 4.

Moreover, 67.8% (95% CI = 61.7-73.8%; n = 155/228) of the Alumni reported specific topics acquired during the EMDM that they perceived helped them in the COVID-19 response. Most of the Alumni reported either the course unit in disaster management or contents related to disaster management (75.48%; n = 117/155). The specific topics that were frequently mentioned were pandemics (20%; n = 31/155), surge capacity (9.3%; n = 14/155), command-control and coordination (5.81%; n = 9), and emergency public health and outbreaks management (3.87%; n = 6). Furthermore, 29.68% of the Alumni (n = 46/155) mentioned EMDM course units or course unit-related contents, other than the disaster management unit, which helped them during the COVID-19 disaster response. In addition, 9.86% of the Alumni (n = 15/155) mentioned soft skills (such as communication, resilience, teamwork, creative thinking, networking, and leadership) that they learned during EMDM were helpful for COVID-19 pandemic response, and 8.93% (n = 13/155) deemed the whole EMDM content helped them during COVID-19 pandemic response.

### Gaps in EMDM Curriculum in Relation to COVID-19 Response

Around 36% (35.96%; 95% CI = 29.9-42.3%; n = 82/228) of the participants deemed some topics were important for the COVID-19 response but were insufficiently or not at all addressed in the EMDM curriculum. Those contents were mainly related to several topics covered in the disaster management course unit (Supplementary Material II; available online only).

## Discussion

A well-trained health professional in disaster management is competent and confident to work under unexpected events such as the COVID-19 pandemic.^24,25^ Unfortunately, the under-representation of disaster medicine in health professional school curricula, and the scarcity of post-graduate disaster medicine education and training programs, left many health care workers under-prepared for the COVID-19 pandemic response.^5,8^

This study demonstrated that the majority of the EMDM Alumni participated in the COVID-19 pandemic response with different roles that varied from policymaking to frontline health care services delivery by working in different settings and at different levels (locally, nationally, and internationally). Interestingly, most of them were requested to change roles increasing the complexity of tasks to be performed and the level of responsibilities. However, few of them reported working beyond their areas of specialization and expertise, unlike many health workers during the current pandemic.^26–28^ Moreover, many Alumni took a leadership role. These findings seem to highlight the relevance of the training they received and display the value in the investment of disaster medicine education making health professionals fit for purpose during disasters.

Effective disaster preparedness and response require interdisciplinary competencies that go beyond disaster and clinical management alone. In fact, disaster medicine professionals are expected to adjust and adapt their professional skills in austere environments and to manage ethical dilemmas of rationing medical care with strained resources.^5,10,29^ Moreover, they should be able to rapidly expand the health response capacity during crises by providing timely training in disaster medicine management that enables other respondents to attain specific required competencies.^5,30^ The findings of this study also showed that, besides disaster management course units and topics, most Alumni acknowledged the usefulness of other EMDM course units. They also valued the networking and soft skills gained through the program to asset COVID-19 response. Still, very few managers and organizations failed to acknowledge these competencies. The administrative and organizational decisions were the most frequent participation barrier reported by the Alumni who did not participate in the COVID-19 response.

The study also revealed gaps in EMDM curriculum that would have been relevant to the COVID-19 pandemic response. For instance, while many participants acknowledged the topics on epidemics and pandemics as useful, still some participants mentioned that the contents were insufficient or suggested specific additional content. This is because the earlier versions of EMDM study program did not contain such contents, and they had been added or updated in the subsequent years. Indeed, some of the mentioned topics need more elaboration or updates, especially for the wide range of roles and responsibilities that Alumni took in the COVID-19 pandemic. The participants also mentioned new topics, which are not included in the EMDM curriculum, such as disaster politics or with new perspectives of disaster management that emerged during COVID-19 such as telemedicine. These new topics may become of interest for disaster medicine specialists due to the specific peculiarities of the COVID-19 pandemic that was unprecedented in its scale and impact and would ultimately reshape the understanding of the disaster medicine science. Nevertheless, these topics should be re-examined carefully for their relevance to inform further review and update of the EMDM curriculum and to provide suggestions for disaster medicine curriculum framework in general.

Finally, the findings of this study demonstrated that the EMDM curriculum had been highly defined in terms of competencies and content, even with the underlined specific content gaps. The study showed that the EMDM curriculum was relevant and helped the Alumni to accomplish various tasks and responsibilities despite the variety of roles they performed during the COVID-19 response. Moreover, the content gaps identified by the Alumni will be used for updating the EMDM curriculum as well as to foster the standardization of the post-graduate disaster medicine curriculum. Therefore, the EMDM curriculum can inform academics and professional bodies interested in developing new academic programs in disaster medicine education to fill the gap in the disaster medicine workforce.

## Limitations

This study relies on Alumni’ self-reported data and their perceived relevance of a post-graduate academic program in disaster medicine such as the EMDM and its curriculum. Hence, the quality or impact of the EMDM program and the Alumni competencies gained through EMDM, or the quality of their response, cannot be evaluated in this study. Nevertheless, the study explored the contribution of disaster medicine specialists during unexpected and large-scale disasters such as the COVID-19 pandemic. The authors acknowledged the limitation associated with the cross-sectional studies, including response bias. However, this study aimed to generate a hypothesis and identify research questions for further research rather than establish evidence. To ensure representative, the sampling frame included all the study population. Moreover, the completion rate was high, and the response rate for the survey was 55.46%, which was above the mean trend of survey response rate (52.7%) reported in the literature,^31^ especially within the context of the COVID-19 pandemic survey fatigue.^32^ Also, recent studies have shown little evidence between response rate and response bias,^33^ particularly for Alumni surveys.^34^

## Conclusions

This study showed that the majority of the EMDM Alumni were involved in the COVID-19 pandemic response playing various roles with an increased level of responsibility compared to those played before the pandemic. Their self-perceived relevance of the EMDM educational program to perform their tasks reaffirmed the value of the investment in disaster medicine academic programs. The identified strengths and gaps in the contents should be an impetus to inform improvement in the EMDM content and provide inputs to standardize the disaster medicine curriculum.

## Supplementary material

For supplementary material accompanying this paper visit https://doi.org/10.1017/S1049023X22001340. click here to view supplementary material
