# Supplementary material for: The Involvement of the European Master in Disaster Medicine (EMDM) Alumni in the COVID-19 Pandemic Response: An Example of the Perceived Relevance of Disaster Medicine Education during Disasters
Source: Prehosp Disaster Med. 2022 Sep 15:1–7. doi: 10.1017/S1049023X22001340 (PMC9530386; doi:10.1017/S1049023X22001340)
Supplement: Supplementary file 1 [file pdmsup.zip › S1049023X22001340sup001.docx]

**Supplementary Material II:**

**Specific contents that Alumni think important for COVID-19 response, but was not addressed by EMDM (n=82)**

| **Course unit** | **Specific topic** | **Specific Content** | **No.** |
| --- | --- | --- | --- |
| **Introduction to disaster medicine** |  |  | **1** |
|  |  | The impact of a global disaster | 1 |
| **Disaster Management** |  |  | **73** |
|  | *Biological emergencies, Pandemics /outbreak response, and /or infectious diseases* |  | 36 |
|  |  | Civil-Military Coordination in Pandemics | 1 |
|  |  | Clinical management | 1 |
|  |  | Infection prevention control (IPC)/ personal protective equipment (PPE) | 3 |
|  |  | International health regulation (IHR) | 1 |
|  |  | Mass isolation and quarantine | 1 |
|  |  | Vaccine (Vaccination/Immunity/ relationship of pharmaceutical industry) | 3 |
|  |  | Virology | 1 |
|  |  | (blank) | 25 |
|  | Communication | (Infodemic, media, Promotion and health education, Risk communication and community engagement (RCCE)) | 8 |
|  | Decision-making and uncertainty |  | 1 |
|  | Economic management |  | 1 |
|  | Health system/ Health services | (Overwhelmed service in the developed world) | 3 |
|  | Hospital management/ Hospital incident command system |  | 2 |
|  | leadership in nursing |  | 1 |
|  | Management of bias |  | 1 |
|  | Planning |  | 1 |
|  | Patient care pathway from the community level up to tertiary level of care |  | 1 |
|  | Public health |  | 2 |
|  | Resilience |  | 1 |
|  | Risk management |  | 1 |
|  | Surge capacity for pandemics | (Hospital layout, IC preparation and mass calculation, patient influx and redistribution) | 8 |
|  | Telehealth |  | 1 |
|  | (blank) |  | 5 |
| **Research in disaster medicine** |  |  | **4** |
|  |  | Fetching right data, Epidemiological statistics and mapping, Surveys, and report | 4 |
| **Mental health** |  |  | **6** |
|  |  | Staff mental health | 2 |
|  |  | (blank) | 4 |
| **Complex Humanitarian Emergencies** |  |  | **3** |
|  |  | Interagency Coordination and the different WHO Coord mechanisms should be expanded in the CHE/ Fragile states | 1 |
|  |  | (blank) | 2 |
| **Legal and ethical aspect of disaster medicine** |  |  | **4** |
|  | Legal aspects of disaster medicine |  | 1 |
|  | Political aspect of disaster medicine | (European solidarity aspects; closing of borders, public relations) | 3 |
| **Live and on-board exercise** |  |  | **1** |
| ***The total number of the content gaps is more than 82 because some respondent reported more than one gap*** | | | |
