# Supplementary material for: The Involvement of the European Master in Disaster Medicine (EMDM) Alumni in the COVID-19 Pandemic Response: An Example of the Perceived Relevance of Disaster Medicine Education during Disasters
Source: Prehosp Disaster Med. 2022 Sep 15:1–7. doi: 10.1017/S1049023X22001340 (PMC9530386; doi:10.1017/S1049023X22001340)
Supplement: Supplementary file 1 [file pdmsup.zip › S1049023X22001340sup002.docx]

**Supplementary Material I: Survey questions**

***Information and consent***

The aim of this questionnaire is to investigate the EMDM alumni role in the response to the Covid-19 pandemic.

The survey is anonymous and voluntary. The expected length is about 10 minutes.

* 1. Do you consent to take part in this survey?

- Yes
- No

***Section 1***

* 2. When did you enrol to the EMDM? (please select the year of enrolment)
2000 2001 2002 2003 2004 2005 2006 2007 2008 2009 2010

2011 2012 2013 2014 2015 2016 2017 2018 2019

* 3. When did you graduate from the EMDM? (please select the year of graduation)
2001 2002 2003 2004 2005 2006 2007 2008 2009 2010

2011 2012 2013 2014 2015 2016 2017 2018 2019 2020 non applicable

* 4. Which was your **main sector** of employment before COVID-19 pandemic?

- Governmental health agency (Ministry of health, regional/district health office)
- Health services delivery (primary, secondary, tertiary, specialized)
- Research/Higher education institution
- United Nations/Non-governmental organization (national/ international)
- Other (please specify): ____________________

* 5. Which was the **level** of your **main** employment before COVID-19 pandemic?

- International level
- National level
- Regional/Provincial level
- District level
- Other (please specify): _________________

* 6. Which of the following better describe your **main** professional **role** before COVID-19 pandemic?

- Physician
- Nurse/ allied health professional
- Public health professional
- Medical education/training
- Managerial role
- Research
- Policy making role
- Other (please specify): ____________

*Either question 6.a or 6.b was shown depending on the answer to question 6*

***6.a. For physicians. What is your specialization?*

- General practice (non-specialized)
- Emergency physician
- Anaesthesiologist
- ICU physician
- Internal medicine specialist
- Infectious diseases physician
- Public health/epidemiology
- Other (please specify) _______________

*** 6.b. For nurses. What is your specialization?*

- Nurse anaesthetist
- Critical care nurse
- Public health/epidemiology
- Other (please specify) ______________

* 7. Have you been directly involved in the COVID-19 response?

- Yes
- No

*For responders who select “no” to question 9, the next question shown is n. 20. Questions from 8 to 19 are shown only to responders who answered “yes” to question 7.*

* 8. In which country did you work for the COVID-19 response?

___________________

* 9. Which of the following better describe your main role in the COVID-19 response?

- Direct COVID-19 patient clinical care
- Epidemiology/outbreak response
- Education/training
- Managerial role
- Research
- Policy making
- Other (please specify) ______________

* 10. Have you changed your **main sector** your employment during the COVID-19 response?

- Yes
- No

** 10.a. If yes, which was the* ***main sector*** *of your employment during COVID-19 response? (question 10.a was shown depending on the answer to question 10)*

- Governmental health agency ( ministry of health, regional/ district health office)
- Health services delivery (primary, secondary and tertiary level)
- Research/ education institution
- UN or Non-governmental (national/ international)
- Other (please specify)

* 11. Have you changed the level of your **main employment** during COVID-19 response?

- Yes
- No

** 11.a. If yes, which was the* ***level*** *of your* ***main employment*** *during COVID-19 response? (question 11.a was shown depending on the answer to question 11)*

- International level
- National level
- Regional/Provincial level
- District level
- Other (please specify)

* 12. Have you changed your **main role** during the COVID-19 pandemic response?

- Yes
- No

** 12.a. If yes, how was the change in your main role? (question 12.a was shown depending on the answer to question 12)*

- *Increase in the level of the tasks and responsibilities*
- Work beyond your main specialization
- Work beyond your main expertise

* 13. What was your working setting during COVID-19 response?

- Intensive care unit
- Sub-intensive care unit
- Infectious unit (ward)
- Medical inpatient ward
- Emergency department
- Pre-hospital care
- Primary care/family physician
- Other (please specify): _____________________

* 14. Which was your degree of decision making in the COVID-19 response?

- I take decisions at organisation level
- I take decisions at unit/sector level
- I take decisions within a small team/related to a
- specific project/area
- I take decisions related to my personal
- activities/operations
- I carry out instructions given by others

***Section 3***

**Please rate how much do you agree with the following statements: (1=strongly disagree, 7=strongly agree)**

* 15. The EMDM helped me managing my tasks in the COVID-19 response

1 2 3 4 5 6 7

* 16. The following outcomes were useful in helping me managing my tasks in the COVID-19 response

- The competences acquired through EMDM 1 2 3 4 5 6 7
- The network of people/organisations created through the EMDM 1 2 3 4 5 6 7

* 17. The following course units and their contents helped me managing my tasks in the COVID-19 response:

- Disaster management 1 2 3 4 5 6 7
- Research in disaster medicine 1 2 3 4 5 6 7
- Mental health in disasters 1 2 3 4 5 6 7
- Education and training 1 2 3 4 5 6 7
- Complex humanitarian emergencies 1 2 3 4 5 6 7
- Ethical aspects 1 2 3 4 5 6 7

* 18. Are there specific topics acquired during the EMDM that are helping you in the COVID-19 response?

If yes, please specify which topics

_______________________________________________________________________

* 19. Are there topics that you find particularly important for the COVID-19 response and were not addressed in the EMDM?

- Yes
- No

* 20. If no, please select the barrier(s) that prevented you from being involved in COVID-19 response (select all that apply): ( the question only shown for responders who select “no” to question 9)

- Personal/family reasons
- Lack of required competence for COVID-19 response
- Organizational/administrative decisions
- Other (please specify)

***Section 3***

21. What is your age?

_______________________

22. What is your gender?

- Female
- Male

* 23. In which country are you currently living?

____________________________

Thank you!

**You answered all the questions, please click "submit response" before leaving this page.**

**Thank you very much for your time!**
